# Deep learning in mental health outcome research: a scoping review

**DOI:** 10.1038/s41398-020-0780-3

**Published:** 2020-04-22

**Authors:** Chang Su, Zhenxing Xu, Jyotishman Pathak, Fei Wang

**Affiliations:** Department of Healthcare Policy and Research, Weill Cornell Medicine, New York, NY USA

**Keywords:** Psychiatric disorders, Biomarkers

## Abstract

Mental illnesses, such as depression, are highly prevalent and have been shown to impact an individual’s physical health. Recently, artificial intelligence (AI) methods have been introduced to assist mental health providers, including psychiatrists and psychologists, for decision-making based on patients’ historical data (e.g., medical records, behavioral data, social media usage, etc.). Deep learning (DL), as one of the most recent generation of AI technologies, has demonstrated superior performance in many real-world applications ranging from computer vision to healthcare. The goal of this study is to review existing research on applications of DL algorithms in mental health outcome research. Specifically, we first briefly overview the state-of-the-art DL techniques. Then we review the literature relevant to DL applications in mental health outcomes. According to the application scenarios, we categorize these relevant articles into four groups: diagnosis and prognosis based on clinical data, analysis of genetics and genomics data for understanding mental health conditions, vocal and visual expression data analysis for disease detection, and estimation of risk of mental illness using social media data. Finally, we discuss challenges in using DL algorithms to improve our understanding of mental health conditions and suggest several promising directions for their applications in improving mental health diagnosis and treatment.

## Introduction

Mental illness is a type of health condition that changes a person’s mind, emotions, or behavior (or all three), and has been shown to impact an individual’s physical health^1,2^. Mental health issues including depression, schizophrenia, attention-deficit hyperactivity disorder (ADHD), and autism spectrum disorder (ASD), etc., are highly prevalent today and it is estimated that around 450 million people worldwide suffer from such problems^1^. In addition to adults, children and adolescents under the age of 18 years also face the risk of mental health disorders. Moreover, mental health illnesses have also been one of the most serious and prevalent public health problems. For example, depression is a leading cause of disability and can lead to an increased risk for suicidal ideation and suicide attempts^2^.

To better understand the mental health conditions and provide better patient care, early detection of mental health problems is an essential step. Different from the diagnosis of other chronic conditions that rely on laboratory tests and measurements, mental illnesses are typically diagnosed based on an individual’s self-report to specific questionnaires designed for the detection of specific patterns of feelings or social interactions^3^. Due to the increasing availability of data pertaining to an individual’s mental health status, artificial intelligence (AI) and machine learning (ML) technologies are being applied to improve our understanding of mental health conditions and have been engaged to assist mental health providers for improved clinical decision-making^4–6^. As one of the latest advances in AI and ML, deep learning (DL), which transforms the data through layers of nonlinear computational processing units, provides a new paradigm to effectively gain knowledge from complex data^7^. In recent years, DL algorithms have demonstrated superior performance in many data-rich application scenarios, including healthcare^8–10^.

In a previous study, Shatte et al.^11^ explored the application of ML techniques in mental health. They reviewed literature by grouping them into four main application domains: diagnosis, prognosis, and treatment, public health, as well as research and clinical administration. In another study, Durstewitz et al.^9^ explored the emerging area of application of DL techniques in psychiatry. They focused on DL in the studies of brain dynamics and subjects’ behaviors, and presented the insights of embedding the interpretable computational models into statistical context. In contrast, this study aims to provide a scoping review of the existing research applying DL methodologies on the analysis of different types of data related to mental health conditions. The reviewed articles are organized into four main groups according to the type of the data analyzed, including the following: (1) clinical data, (2) genetic and genomics data, (3) vocal and visual expression data, and (4) social media data. Finally, the challenges the current studies faced with, as well as future research directions towards bridging the gap between the application of DL algorithms and patient care, are discussed.

## Deep learning overview

ML aims at developing computational algorithms or statistical models that can automatically infer hidden patterns from data^12,13^. Recent years have witnessed an increasing number of ML models being developed to analyze healthcare data^4^. However, conventional ML approaches require a significant amount of feature engineering for optimal performance—a step that is necessary for most application scenarios to obtain good performance, which is usually resource- and time-consuming.

**Fig. 1:**
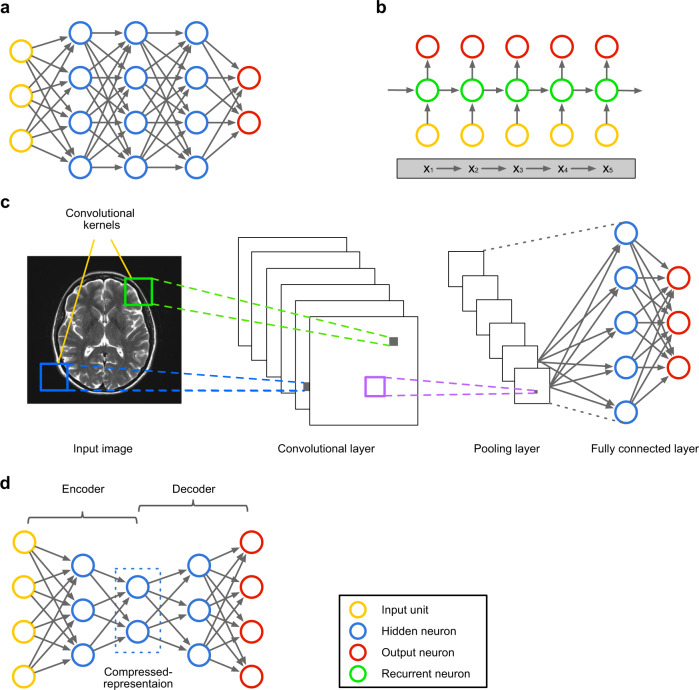
Examples of deep neural networks. **a** Deep feedforward neural network (DFNN). It is the basic design of DL models. Commonly, a DFNN contains multiple hidden layers. **b** A recurrent neural network (RNN) is presented to process sequence data. To encode history information, each recurrent neuron receives the input element and the state vector of the predecessor neuron, and yields a hidden state fed to the successor neuron. For example, not only the individual information but also the dependence of the elements of the sequence x_1_ → x_2_ → x_3_ → x_4_ → x_5_ is encoded by the RNN architecture. **c** Convolutional neural network (CNN). Between input layer (e.g., input neuroimage) and output layer, a CNN commonly contains three types of layers: the convolutional layer that is to generate feature maps by sliding convolutional kernels in the previous layer; the pooling layer is used to reduce dimensionality of previous convolutional layer; and the fully connected layer is to make prediction. For the illustrative purpose, this example only has one layer of each type; yet, a real-world CNN would have multiple convolutional and pooling layers (usually in an interpolated manner) and one fully connected layer. **d** Autoencoder consists of two components: the encoder, which learns to compress the input data into a latent representation layer by layer, whereas the decoder, inverse to the encoder, learns to reconstruct the data at the output layer. The learned compressed representations can be fed to the downstream predictive model.

As the newest wave of ML and AI technologies, DL approaches aim at the development of an end-to-end mechanism that maps the input raw features directly into the outputs through a multi-layer network structure that is able to capture the hidden patterns within the data. In this section, we will review several popular DL model architectures, including deep feedforward neural network (DFNN), recurrent neural network (RNN)^14^, convolutional neural network (CNN)^15^, and autoencoder^16^. Figure 1 provides an overview of these architectures.

### Deep feedforward neural network

**Fig. 2:**
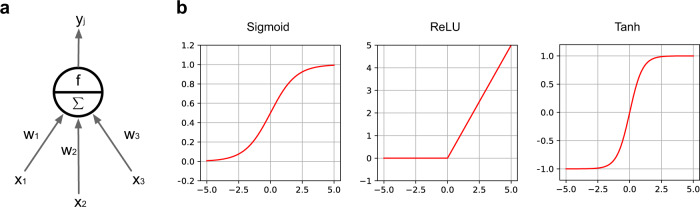
Technical details of neural networks. **a** An illustration of basic unit of neural networks, i.e., artificial neuron. Each input *x*_*i*_ is associated with a weight *w*_*i*_. The weighted sum of all inputs Σ*w*_*i*_*x*_*i*_ is fed to a nonlinear activation function *f* to generate the output *y*_*j*_ of the *j*-th neuron, i.e., *y*_*j*_ = *f*(Σ*w*_*i*_*x*_*i*_). **b** Illustrations of the widely used nonlinear activation function.

Artificial neural network (ANN) is proposed with the intention of mimicking how human brain works, where the basic element is an artificial neuron depicted in Fig. 2a. Mathematically, an artificial neuron is a nonlinear transformation unit, which takes the weighted summation of all inputs and feeds the result to an activation function, such as sigmoid, rectifier (i.e., rectified linear unit [ReLU]), or hyperbolic tangent (Fig. 2b). An ANN is composed of multiple artificial neurons with different connection architectures. The simplest ANN architecture is the feedforward neural network (FNN), which stacks the neurons layer by layer in a feedforward manner (Fig. 1a), where the neurons across adjacent layers are fully connected to each other. The first layer of the FNN is the input layer that each unit receives one dimension of the data vector. The last layer is the output layer that outputs the probabilities that a subject belonging to different classes (in classification). The layers between the input and output layers are the hidden layers. A DFNN usually contains multiple hidden layers. As shown in Fig. 2a, there is a weight parameter associated with each edge in the DFNN, which needs to be optimized by minimizing some training loss measured on a specific training dataset (usually through backpropagation^17^). After the optimal set of parameters are learned, the DFNN can be used to predict the target value (e.g., class) of any testing data vectors. Therefore, a DFNN can be viewed as an end-to-end process that transforms a specific raw data vector to its target layer by layer. Compared with the traditional ML models, DFNN has shown superior performance in many data mining tasks and have been introduced to the analysis of clinical data and genetic data to predict mental health conditions. We will discuss the applications of these methods further in the Results section.

#### Recurrent neural network

RNNs were designed to analyze sequential data such as natural language, speech, and video. Given an input sequence, the RNN processes one element of the sequence at a time by feeding to a recurrent neuron. To encode the historical information along the sequence, each recurrent neuron receives the input element at the corresponding time point and the output of the neuron at previous time stamp, and the output will also be provided to the neuron at next time stamp (this is also where the term “recurrent” comes from). An example RNN architecture is shown in Fig. 1b where the input is a sequence of words (a sentence). The recurrence link (i.e., the edge linking different neurons) enables RNN to capture the latent semantic dependencies among words and the syntax of the sentence. In recent years, different variants of RNN, such as long short-term memory (LSTM)^18^ and gated recurrent unit^19^ have been proposed, and the main difference among these models is how the input is mapped to the output for the recurrent neuron. RNN models have demonstrated state-of-the-art performance in various applications, especially natural language processing (NLP; e.g., machine translation and text-based classification); hence, they hold great premise in processing clinical notes and social media posts to detect mental health conditions as discussed below.

#### Convolutional neural network

CNN is a specific type of deep neural network originally designed for image analysis^15^, where each pixel corresponds to a specific input dimension describing the image. Similar to a DFNN, CNN also maps these input image pixels to the corresponding target (e.g., image class) through layers of nonlinear transformations. Different from DFNN, where only fully connected layers are considered, there are typically three types of layers in a CNN: a convolution–activation layer, a pooling layer, and a fully connected layer (Fig. 1c). The convolution–activation layer first convolves the entire feature map obtained from previous layer with small two-dimensional convolution filters. The results from each convolution filter are activated through a nonlinear activation function in the same way as a DFNN. A pooling layer reduces the size of the feature map through sub-sampling. The fully connected layer is analogous to the hidden layer in a DFNN, where each neuron is connected to all neurons of the previous layer. The convolution–activation layer extracts locally invariant patterns from the feature maps. The pooling layer effectively reduces the feature dimensionality to avoid model overfitting. The fully connected layer explores the global feature interactions as in DFNNs. Different combinations of these three types of layers constitute different CNN architectures. Because of the various characteristics of images such as local self-similarity, compositionality, and translational and deformation invariance, CNN has demonstrated state-of-the-art performance in many computer vision tasks^7^. Hence, the CNN models are promising in processing clinical images and expression data (e.g., facial expression images) to detect mental health conditions. We will discuss the application of these methods in the Results section.

#### Autoencoder

Autoencoder is a special variant of the DFNN aimed at learning new (usually more compact) data representations that can optimally reconstruct the original data vectors^16,20^. An autoencoder typically consists of two components (Fig. 1d) as follows: (1) the encoder, which learns new representations (usually with reduced dimensionality) from the input data through a multi-layer FNN; and (2) the decoder, which is exactly the reverse of the encoder, reconstructs the data in their original space from the representations derived from the encoder. The parameters in the autoencoder are learned through minimizing the reconstruction loss. Autoencoder has demonstrated the capacity of extracting meaningful features from raw data without any supervision information. In the studies of mental health outcomes, the use of autoencoder has resulted in desirable improvement in analyzing clinical and expression image data, which will be detailed in the Results section.

## Methods

The processing and reporting of the results of this review were guided by the Preferred Reporting Items for Systematic Reviews and Meta-Analyses guidelines^21^. To thoroughly review the literature, a two-step method was used to retrieve all the studies on relevant topics. First, we conducted a search of the computerized bibliographic databases including PubMed and Web of Science. The search strategy is detailed in Supplementary Appendix 1. The literature search comprised articles published until April 2019. Next, a snowball technique was applied to identify additional studies. Furthermore, we manually searched other resources, including Google Scholar, and Institute of Electrical and Electronics Engineers (IEEE Xplore), to find additional relevant articles.

**Fig. 3:**
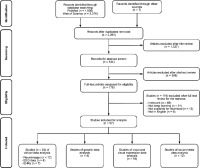
PRISMA flow diagram: deep learning in mental health outcome research. In total, 57 studies, in terms of clinical data analysis, genetic data analysis, vocal and visual expression data analysis, and social media data analysis, which met our eligibility criteria, were included in this review.

Figure 3 presents the study selection process. All articles were evaluated carefully and studies were excluded if: (1) the main outcome is not a mental health condition; (2) the model involved is not a DL algorithm; (3) full-text of the article is not accessible; and (4) the article is written not in English.

## Results

A total of 57 articles met our eligibility criteria. Most of the reviewed articles were published between 2014 and 2019. To clearly summarize these articles, we grouped them into four categories according to the types of data analyzed, including (1) clinical data, (2) genetic and genomics data, (3) vocal and visual expression data, and (4) social media data. Table 1 summarizes the characteristics of these selected studies.

**Table 1:** A summary of the selected studies in this review.

Authors, year	Used deep model	Data	Study cohort	Outcome assessment	Aims	Performance	Findings
Clinical data							
Neuroimage data							
Kuang and He., 2014^25^	DBN	fMRI	449 Subjects (ADHD-200^a^)	Human annotation	Prediction of ADHD status and subtype	ACC = 0.407–0.809	The model is the first time that the DL method has been used for the discrimination of ADHD with fMRI data.
Kuang et al., 2014^26^	DBN	fMRI	492 Subjects (ADHD-200^a^)	Human annotation	Prediction of ADHD status and subtype	ACC = 0.344–0.718	The study verified that there is difference between ADHD and control in the prefrontal cortex and cingulated cortex.
Ulloa et al., 2015^35^	DFNN	sMRI	198 Schizophrenia subjects, 191 HCs	Human annotation	Prediction of schizophrenia	ACC = 0.75; Baseline ACC = 0.70	The model classified neuroimaging data in an online fashion using purely synthetic data.
Pinaya et al., 2016^33^ (source code available^k^)	DBN	sMRI	143 Schizophrenia subjects, 32 first-episode psychosis, 191 HCs	Human annotation based on SCID-I	Prediction of schizophrenia	ACC = 0.736; Baseline ACC = 0.681	The DBN highlighted differences between classes, especially in the frontal, temporal, parietal, and insular cortices, and in some subcortical regions, including the corpus callosum, putamen, and cerebellum.
Farzi et al., 2017^27^	DBN	fMRI	336 Subjects (ADHD-200^a^)	Human annotation	Prediction of ADHD	ACC = 0.637–0.698; Baseline ACC = 0.352–0.642	The deep model captured relationships from multiple features, including FMRI features, diagnosis status, ADHD measures, secondary symptoms, age, gender, etc.
Zou et al., 2017^28^	3D CNN	fMRI	239 ADHDs, 429 TDCs	Human annotation	Prediction of ADHD	ACC = 0.657; Baseline ACC = 0.615	The 3D CNN architecture can detect physiologically meaningful 3D local patterns from fMRI data.
Geng and Xu, 2017^37^	Autoencoder and CNN	fMRI	24 MDDs, 24 HCs	Not specified	Prediction of depression	ACC = 0.95; Baseline ACC = 0.71	The model automatically learned meaningful features from the origin time series of the fMRI.
Zou et al., 2017^30^	3D CNN	fMRI and sMRI	239 ADHDs, 429 TDCs	Human annotation	Prediction of ADHD	ACC = 0.692; Baseline ACC = 0.615	The study found that brain functional and structural information are complementary. The low-level features and high-level features from fMRI and sMRI are useful for the detection of ADHD.
Sen et al., 2018^31^	Autoencoder	fMRI and sMRI	279 ADHDs, 491 HCs (ADHD-200^a^); 538 ASDs and 573 HCs (ABIDE^b^)	Human annotation	Prediction of ADHD and ASD	ACC = 0.643–0.673; Baseline ACC = 0.500–0.516	Combining multimodal features can yield good classification accuracy for diagnosis of ADHD and autism.
Aghdam et al., 2018^38^	DBN	fMRI and sMRI	116 ASDs, 69 TDCs (ABIDE ^b^)	Human annotation	Prediction of ASD	ACC = 0.656	(1) There were significant relationships between rs-fMRI and sMRI; (2) Increasing the depth of DBN can help improve diagnostic classification.
Matsubara et al., 2019^36^	DFNN	fMRI	50 Schizophrenia subjects, 49 BDs, and 122 HCs^c^		Diagnosis of psychiatric disorder	ACC = 0.766; Baseline ACC = 0.720	The study modeled joint distribution of rs-fMRI data, class labels, and remaining frame-wise variabilities.
Pinaya et al., 2019^34^	Autoencoder	sMRI	35 Schizophrenia subjects, 40 HCs (NUSDAST^d^); 83 ASDs, 105 HCs (ABIDE^b^)	Human annotation	Identification of abnormal brain structural patterns in neuropsychiatric disorders (schizophrenia and ASD)	ACC = 0.639 to 0.707; Baseline ACC = 0.569 to 0.637	There are distinct patterns of neuroanatomical deviations for the two diseases (schizophrenia and ASD).
Electroencephalogram data							
Mohan et al., 2017^44^	DFNN	6.25-sec EEG	116 University students	PHQ-9 score and DASS-21	Prediction of depression	19 Out of 20 testers were detected correctly	The profound outcome of this study showed the signals collected from central (C3 and C4) region are marginally higher compared other brain regions.
Acharya et al., 2018^43^	CNN	5-min EEG	15 Depressed subjects, 15 HCs	Human annotation based on specific questions and physical examination	Prediction of depression	ACC = 0.935 (left hemisphere) and 0.960 (right hemisphere)	The study found that the EEG signals from the right hemisphere are more distinctive in depression than those from the left hemisphere.
Zhang et al., 2018^45^	CNN	1000 Hz EEG	20 subjects	Cross-task mental workload assessment	Cross-task mental workload assessment	ACC = 0.889	(1) Spectral changes of EEG hemispheric asymmetry provide effective information to distinguish different mental workload tasks. (2) Different time periods can provide different hemispheric EEG activities, and selection of an appropriate time window is essential for extracting hemispheric asymmetry information.
Li et al., 2019^46^	CNN	1-sec EEG	24 Mild depression, 24 HCs	BDI-II	Prediction of mild depression	ACC = 0.856	They found that the spectral information of EEG played a major role and the temporal information of EEG provided a statistically significant improvement to accuracy.
Electronic health records							
Pham et al., 2017^51^ (source code available ^l^)	DeepCare (LSTM based model)	Longitudinal EMRs	11,000 Patients	ICD-10 diagnosis code	Prediction of the future mental outcomes	F-score = 0.754; Baseline F-score = 0.679	The LSTM architecture appropriately captured disease progression by modeling the illness history but also the medical interventions.
Geraci et al., 2017^53^	DFNN	Clinical notes	366 Patients	Human annotation	Prediction of youth depression	Sensitivity = 0.935, Specificity = 0.68, Positive predictive value = 0.77	The model identified individuals who meet the inclusion–exclusion criteria for depression research.
Rios and Kavuluru, 2017^56^	CNN	Clinical notes	1000 Neuropsychiatric notes^e^	Human annotation	Prediction of psychiatric symptom severity	NMMAE = 0.856	The CNN scheme showed superiority in extract text features and the predictive performance is better than many traditional text classification methods.
Tran and Kavuluru, 2017^58^	CNN and attention-based RNN	Clinical notes	1000 Neuropsychiatric notes^e^	Human annotation	Prediction of 11 mental health conditions (e.g., ADHD, anxiety, bipolar, dementia, depression, etc.)	F-score = 0.631; Baseline F-score = 0.598	Both the CNN and RNN architectures achieved desirable prediction performances.
Choi et al., 2018^50^	DFNN	Structured EHRs	SD: 2546, HC: 817,405	ICD-10 diagnosis code	Prediction of suicide death	AUC = 0.683; Baseline AUC = 0.688	The model is able to address the imbalance classification problem.
Lin et al., 2018^52^	DFNN	Clinical biomarkers and genetic biomarkers (SNPs)	257 MDD treatment responders, 164 MDD treatment non-responders	HRSD	Prediction of antidepressant response and remission	AUC = 0.823; Baseline AUC = 0.816	The model achieved better performance than the logistic regression classifier.
Dai and Jonnagaddala, 2018^57^	CNN	Clinical notes	Clinical notes of psychiatric disorder subjects: Absent: 92, Mild: 252, Moderate: 156, Severe: 149^e^	Human annotation	Prediction of positive valence symptom severity	MAE = 0.539; Baseline MAE = 0.583	The CNN models provided comparable solutions without sophisticated preprocessing on the text data.
Genetic data							
Laksshman et al., 2017^71^	CNN	Whole exome sequencing data	1000 Subjects^f^	Not specified	Differentiating bipolar disorder patients with healthy controls	AUC = 0.65; Baseline AUC = 0.62	The 1D convolution captured correlation of neighboring loci. The model achieved a winning predictive performance of 0.65 AUC, compared with traditional methods ranging from 0.5 to 0.55. This revealed that the model might be picking up complex patterns across the samples.
Khan and Wang, 2017^67^ (source code available^m^)	ncDeepBrain (DFNN based)	Genome sequencing data	-	Not specified	Identification of non-coding variants associated with mental disorders	ACC = 0.82; Baseline ACC = 0.71	The model was trained for scoring the non-coding variants for prioritization.
Khan et al., 2018^68^ (source code available^m^)	iMEGES (DFNN based)	Genome sequencing data	-	Not specified	Prioritization of susceptibility genes for mental disorders	AUC = 0.57 (schizophrenia) and 0.58 (ASD)	The model integrated the ncDeepBrain score, general gene scores, and disease-specific scores to prioritize susceptibility genes for mental disorders.
Wang et al., 2018^69^	Deep structured phenotype network (DSPN)	Regulatory network	PsychENCODE Consortium dataset ^g^	Not specified	Prediction of psychiatric phenotypes from genotype and expression	ACC = 0.729; Baseline ACC = 0.681	The model provided insights about intermediate phenotypes and their connections to high-level phenotypes (disease traits).
Vocal and visual expression data							
Chao et al., 2015^84^	CNN and LSTM	Voice and visual data	84 Subjects (AVEC dataset)	Human annotation	Prediction of depression severity	MAE = 8.7	Face appearance features were extracted by CNN. The deep-learned appearance features, combined with audio and face shape features, were fed to a LSTM to capture long-term sequence features.
Yang et al., 2016^85^	LSTM and autoencoder	Elicited speech voice data	13 BDs, 13 UDs, and 13 HCs (Chi-Mei mood dataset)	Human annotation	Prediction of mood disorder	ACC = 0.769; Baseline ACC = 0.498	The denoising autoencoder adopted emotion domain data to the speech data space to generate emotion profiles (EPs). The LSTM characterized the temporal evolution of the EP sequence with respect to eliciting emotional videos.
Ma et al., 2016^76^	CNN and LSTM	Voice data	(AVEC dataset)	PHQ-8 score	Prediction of depression	F-score = 0.52	The model incorporated short-term temporal and spectral correlations by a 1D CNN, captured middle-term correlations by 1D max-pooling, and extracted long-term correlations with LSTM.
Huang et al., 2017^86^	LSTM and autoencoder	Elicited speech voice data	15 BDs, 15 UDs, and 15 HCs (Chi-Mei mood dataset)	Human annotation	Prediction of mood disorder	ACC = 0.733	The denoising autoencoder adopted emotion domain data to the speech data space to generate emotion profiles (EPs). The LSTM characterized the temporal evolution of the EP sequence with respect to eliciting emotional videos.
Su et al., 2017^90^	LSTM and autoencoder	Elicited video data	12 BDs, 12 MDDs, and 12 HCs (Chi-Mei mood dataset)	Human annotation	Classification of mood disorders	ACC = 0.677; Baseline ACC = 0.556	The study modeled the long-term variation among different mood disorders types by LSTM.
Jaiswal et al., 2017^92^	CNN	Facial expression RGBD data (A RGB-D image is simply a combination of a color image and its corresponding depth image.)	4 ADHDs, 22 ASDs, 11 ADHD + ASDs, and 18 HCs	Not specified	Prediction of ADHD and ASD	ACC = 0.96 (condition vs. HC) and 0.93 (ADHD + ASD vs. ASD only)	The study established the relationship between facial expression/gestures and neurodevelopmental conditions such as ADHD and ASD.
Cho et al., 2017^93^ (source code available^n^)	CNN	Thermal images	8 Healthy adults^h^	Human annotation	Recognition of psychological stress level (mental overload)	ACC = 0.846 (no stress vs. stress) and 0.565 (no stress vs. low-level stress vs. high-level stress)	The model identified psychological stress level by using a low-cost thermal camera, which tracks the person’s breathing patterns.
Yang et al., 2017^79^	CNN and DFNN	Voice and visual data	189 Segments of clinical interview (AVEC dataset)	PHQ-8 score	Prediction of depression	MAE = 5.4	The study proposed a multimodal approach: two CNNs were introduced to encode audio and video data, respectively. Then a fully connected DNN was used to combine the two channel feature maps to predict PHQ-8 scores.
Gupta et al., 2017^94^	DFNN	Voice and visual data	300 Video samples (AVEC dataset)	Valence, arousal, and dominance ratings by human annotation	Affective prediction	Correlation coefficient *ρ* between the true and predicted ratings: 0.21–0.51	The DFNN incorporated depression severity as the parameter, linking the effects of depression on subjects’ affective expressions.
He and Cao, 2018^77^	CNN	Voice data	300 Video samples (AVEC dataset)	BDI-II	Prediction of depression	MAE = 8.2; Baseline MAE = 10.4	The model consists of four CNNs, one for extracting audio features from raw waveform, one for extracting texture features from spectrogram images, and two for modeling handcraft features.
Dawood et al., 2018^81^	CNN and LSTM	Video collected by webcam	862 Videos of AS, 545 videos of TDC	Not specified	Prediction of depression	ACC = 0.901	The model takes the power of CNN to learn facial expression features from images (frame’s response map) and LSTM to learn from series of temporal data (sequence of response maps).
Song et al., 2018^82^	CNN	Video data	30 Depressed subjects, 77 non-depressed subjects, and 35 subjects for development (AVEC dataset)	PHQ-8 score	Prediction of depression and depression severity	MAE = 5.01; Baseline = 4.4	The model transformed behavior signals to spectrum maps to capture long-term series information. Then CNN was used to extract spectral features.
Zhu et al., 2018^83^	CNN	Video data	340 Videos from 292 subjects (AVEC dataset)	BDI-II	Prediction of depression	MAE = 7.6; Baseline MAE = 8.2	The model introduced two CNNs, one pre-trained for modeling the static facial appearance and the other modeling the optical flow images extracted from different frames.
Prasetio et al., 2018^91^	CNN	Facial image	Female: 87 high stress, 129 low stress, and 175 neutral; Male: 134 high stress, 212 low stress, and 237 neutral	Human annotation	Stress recognition	ACC = 0.959; Baseline ACC = 0.890	The features were from facial images and fed to a CNN to identify stress.
Jan et al. 2018^87^	CNN (only for image	Voice and visual data	300 Videos (AVEC dataset)	BDI-II	Prediction of depression severity	MAE = 6.7 (Unimodal) and 6.1 (Bimodal); Baseline MAE = 8.0 (Unimodal) and 6.4 (Bimodal)	The deep-learnt features showed significant improvement on prediction.
Harati et al. 2018^89^	LSTM	Audio of interview during Deep Brain Stimulation treatment	13 Subjects	HRMD score	Prediction of depression severity	AUC = 0.80	The model extracted emotion features from patients’ clinical audio utterances.
Huang et al. 2019^80^	CNN and LSTM	Elicited speech voice data	15 BDs, 15 UDs, and 15 HCs (Chi-Mei mood dataset)	Human annotation	Short-term detection of mood disorders	ACC = 0.756; Baseline ACC = 0.622	The CNN was used to generate an emotion profile (EP) of each elicited speech response. The LSTM was used to characterize temporal evolution of EPs of patients
Su et al., 2019^88^	Autoencoder and LSTM	Voice and visual data	13 BDs, 13 UDs, and 13 HCs (Chi-Mei mood dataset)	Human annotation	Prediction of mood disorder	ACC = 0.692; Baseline ACC = 0.498	Autoencoder generated bottleneck features of the facial expression and speech response. LSTM modeled the temporal information of all elicited responses. The model is able to overcome misdiagnosis of bipolar disorder as unipolar disorder.
Social media data							
Lin et al., 2014^98^	CNN and DFNN	Sina Weibo posts	11,074 Subjects of stress, 12,230 subjects of no stress	Pattern matching in tweets	Stress detection	ACC = 0.756–0.844	There are relationships between users’ stress and their tweeting content, social engagement, and behavior patterns.
Lin et al., 2014^99^	Denoising autoencoder	Hashtag-labeled tweets	3634 Tweets of affection stress, 3966 tweets of work stress, 5747 tweets with social stress, 13,973 tweets of physiological stress, 14,543 tweets of other stress, and 14,931 tweets of no stress	User-labeled hashtag	Stress detection	ACC = 0.823; Baseline ACC = 59.7	Detection results were improved by using deep neural network models.
Gkotsis et al., 2017^106^	CNN and DFNN	Reddit posts	538,245 Posts related to 11 mental themes, 476,388 non-mental health posts^i^	Human annotation	Identification of posts related to mental illness	ACC = 0.911 (binary classification) and 0.714 (multiclass classification); Baseline ACC = 0.908 (binary classification) and 0.708 (multiclass classification)	(1) The most common misclassification is depression; (2) Some of the themes are highly inter-related and not always distinguishable as separate and exclusive classes.
Li et al., 2017^100^	RNN	Tencent Weibo posts	29,232 Posts of 124 students, containing 122 study-related stressor events	Human annotation	Prediction of adolescent stress	MSE = 0.19; Baseline MSE = 0.25	The model incorporated relationships of stressor events and improved the prediction of stress in adolescent.
Lin et al., 2017^101^	CNN	Sina Weibo posts, Tencent Weibo posts, and Twitter posts; social interactions	11,074 Subjects of stress, 12,230 subjects of no stress	Pattern matching in tweets	Stress detection	ACC = 0.916	Users stress state is closely related to that of his/her friends in social media.
Sadeque et al., 2017^104^	GRU	Reddit posts	136 Depressed subjects, 752 HCs	Self-declaration of depression in posts	Prediction of early depression	F-score = 0.64; Baseline F-score = 0.40	The RNN captured sequential information from texts with sequential property.
Cong et al., 2018^102^	LSTM	The Reddit Self-reported Depression Diagnosis (RSDD) dataset	9000 Depressed subjects, 107,000 HCs	Self-declaration of depression in posts	Prediction of depression	F-score = 0.60; Baseline F-score = 0.44	The model reduced data imbalance and enhanced classification capacity.
Coppersmith et al., 2018^107^	LSTM	Social media posts	418 Users with suicide attempts; number of HC not specified	Self-declaration of depression in posts	Prediction of suicidal risk	AUC = 0.94	The LSTM captured contextual information between words and better obtained nuances of language related to mental health.
Du et al, 2018^108^	CNN and RNN	Twitter posts	1,962,766 Tweets	Suicide-related keywords matching	Identification of suicide-related psychiatric stressors	ACC = 0.74 (CNN) and 0.72 (RNN); Baseline ACC = 0.703	CNN- and RNN-based model obtained better performance at identifying suicide-related tweets and psychiatric stressors, respectively.
Ive et al., 2018^103^	GRU	Social media posts	538,245 Posts related to 11 mental themes, 476,388 non-mental health posts	Human annotation	Classification of media text related to mental health	ACC = 0.76	RNN has the intrinsic ability of considering input in its sequence and the hierarchical structure is beneficial for the analysis of health-related online text.
Fraga et al., 2018^105^	RNN	Reddit posts	261,511 Posts and 1,256,669 comments from 105,878 users related to depression, 44,541 users related to SuicideWatch, 43,321 users related to anxiety, 13,939 users related to BD^j^	Keywords matching	Analysis of four subreddits (anxiety, bipolar, depression, and suicide) related to mental health disorders	–	(1) Interaction patterns are very similar across the subreddits and interactions are centered around content rather than users; (2) the four subreddits share a common language.
Alambo et al., 2019^109^	RNN	Reddit posts	4992 Posts of 500 users	Human annotation	Prediction of suicidal risk	–	This study generated a gold standard dataset of suicide posts with their risk levels and formed a basis for the next step of constructing conversational agents that elicited suicide-related natural conversation on basis of questions.

*ACC* accuracy, *ADHD* attention-deficit hyperactivity disorder, *ASD* autism spectrum disorder, *AUC* area under the receiver operating characteristic curve, *AVEC* Audio-Visual Emotion recognition Challenge, *BD* bipolar disorder, *BDI-II* Beck Depression Inventory II, *CNN* convolutional neural network, *DASS-21* Depression Anxiety stress scale, *DBN* deep belief network, *DFNN* deep feedforward neural network, *GRU* gated recurrent unit network, *HC* healthy control, *HRSD* Hamilton Rating Scale for Depression, *LSTM* long short-term memory network, *MAE* mean absolute error, *MSE* mean squared error, *NMMAE* normalized macro mean absolute error, *PHQ-8* Patient Health Questionnaire eighth version, *PHQ-9* Patient Health Questionnaire ninth version, *RNN* recurrent neural network, *SCID-I* Structured Clinical Interview for DSM-IV, *SNP* single-nucleotide polymorphism, *TDC* typical developing control, *UD* unipolar depression

^a^ADHD-200 dataset, http://fcon_1000.projects.nitrc.org/indi/adhd200/

^b^ABIDE dataset, http://fcon_1000.projects.nitrc.org/indi/abide/

^c^https://openneuro.org/datasets/ds000030

^d^NUSDAST dataset, http://schizconnect.org

^e^https://www.i2b2.org/NLP/RDoCforPsychiatry/

^f^https://genomeinterpretation.org/content/4-bipolar-exomes

^g^PsychENCODE Consortium dataset, https://www.nimhgenetics.org/resources/psychencode

^h^http://youngjuncho.com/datasets/

^i^https://www.reddit.com/comments/3mg812

^j^http://files.pushshift.io/reddit/

^k^https://github.com/mihaelacr/pydeeplearn

^l^https://github.com/trangptm/DeepCare

^m^https://github.com/WGLab/iMEGES

^n^http://youngjuncho.com/2017/acii2017-open-sources/

### Clinical data

#### Neuroimages

Previous studies have shown that neuroimages can record evidence of neuropsychiatric disorders^22,23^. Two common types of neuroimage data analyzed in mental health studies are functional magnetic resonance imaging (fMRI) and structural MRI (sMRI) data. In fMRI data, the brain activity is measured by identification of the changes associated with blood flow, based on the fact that cerebral blood flow and neuronal activation are coupled^24^. In sMRI data, the neurological aspect of a brain is described based on the structural textures, which show some information in terms of the spatial arrangements of voxel intensities in 3D. Recently, DL technologies have been demonstrated in analyzing both fMRI and sMRI data.

One application of DL in fMRI and sMRI data is the identification of ADHD^25–31^. To learn meaningful information from the neuroimages, CNN and deep belief network (DBN) models were used. In particular, the CNN models were mainly used to identify local spatial patterns and DBN models were to obtain a deep hierarchical representation of the neuroimages. Different patterns were discovered between ADHDs and controls in the prefrontal cortex and cingulated cortex. Also, several studies analyzed sMRIs to investigate schizophrenia^32–36^, where DFNN, DBN, and autoencoder were utilized. These studies reported abnormal patterns of cortical regions and cortical–striatal–cerebellar circuit in the brain of schizophrenia patients, especially in the frontal, temporal, parietal, and insular cortices, and in some subcortical regions, including the corpus callosum, putamen, and cerebellum. Moreover, the use of DL in neuroimages also targeted at addressing other mental health disorders. Geng et al.^37^ proposed to use CNN and autoencoder to acquire meaningful features from the original time series of fMRI data for predicting depression. Two studies^31,38^ integrated the fMRI and sMRI data modalities to develop predictive models for ASDs. Significant relationships between fMRI and sMRI data were observed with regard to ASD prediction.

##### Challenges and opportunities

The aforementioned studies have demonstrated that the use of DL techniques in analyzing neuroimages can provide evidence in terms of mental health problems, which can be translated into clinical practice and facilitate the diagnosis of mental health illness. However, multiple challenges need to be addressed to achieve this objective. First, DL architectures generally require large data samples to train the models, which may pose a difficulty in neuroimaging analysis because of the lack of such data^39^. Second, typically the imaging data lie in a high-dimensional space, e.g., even a 64 × 64 2D neuroimage can result in 4096 features. This leads to the risk of overfitting by the DL models. To address this, most existing studies reported to utilize MRI data preprocessing tools such as Statistical Parametric Mapping (https://www.fil.ion.ucl.ac.uk/spm/), Data Processing Assistant for Resting-State fMRI^40^, and fMRI Preprocessing Pipeline^41^ to extract useful features before feeding to the DL models. Even though an intuitive attribute of DL is the capacity to learn meaningful features from raw data, feature engineering tools are needed especially in the case of small sample size and high-dimensionality, e.g., the neuroimage analysis. The use of such tools mitigates the overfitting risk of DL models. As reported in some selected studies^28,31,35,37^, the DL models can benefit from feature engineering techniques and have been shown to outperform the traditional ML models in the prediction of multiple conditions such as depression, schizophrenia, and ADHD. However, such tools extract features relying on prior knowledge; hence may omit some information that is meaningful for mental outcome research but unknown yet. An alternative way is to use CNN to automatically extract information from the raw data. As reported in the previous study^10^, CNNs perform well in processing raw neuroimage data. Among the studies reviewed in this study, three^29,30,37^ reported to involve CNN layers and achieved desirable performances.

#### Electroencephalogram data

As a low-cost, small-size, and high temporal resolution signal containing up to several hundred channels, analysis of electroencephalogram (EEG) data has gained significant attention to study brain disorders^42^. As the EEG signal is one kind of streaming data that presents a high density and continuous characteristics, it challenges traditional feature engineering-based methods to obtain sufficient information from the raw EEG data to make accurate predictions. To address this, recently the DL models have been employed to analyze raw EEG signal data.

Four articles reviewed proposed to use DL in understanding mental health conditions based on the analysis of EEG signals. Acharya et al.^43^ used CNN to extract features from the input EEG signals. They found that the EEG signals from the right hemisphere of the human brain are more distinctive in terms of the detection of depression than those from the left hemisphere. The findings provided shreds of evidence that depression is associated with a hyperactive right hemisphere. Mohan et al.^44^ modeled the raw EEG signals by DFNN to obtain information about the human brain waves. They found that the signals collected from the central (C3 and C4) regions are marginally higher compared with other brain regions, which can be used to distinguish the depressed and normal subjects from the brain wave signals. Zhang et al.^45^ proposed a concatenated structure of deep recurrent and 3D CNN to obtain EEG features across different tasks. They reported that the DL model can capture the spectral changes of EEG hemispheric asymmetry to distinguish different mental workload effectively. Li et al.^46^ presented a computer-aided detection system by extracting multiple types of information (e.g., spectral, spatial, and temporal information) to recognize mild depression based on CNN architecture. The authors found that both spectral and temporal information of EEG are crucial for prediction of depression.

##### Challenges and opportunities

EEG data are usually classified as streaming data that are continuous and are of high density. Despite the initial success in applying DL algorithms to analyze EEG data for studying multiple mental health conditions, there exist several challenges. One major challenge is that raw EEG data gathered from sensors have a certain degree of erroneous, noisy, and redundant information caused by discharged batteries, failures in sensor readings, and intermittent communication loss in wireless sensor networks^47^. This may challenge the model in extracting meaningful information from noise. Multiple preprocessing steps (e.g., data denoising, data interpolation, data transformation, and data segmentation) are necessary for dealing with the raw EEG signal before feeding to the DL models. Besides, due to the dense characteristics in the raw EEG data, analysis of the streaming data is computationally more expensive, which poses a challenge for the model architecture selection. A proper model should be designed relatively with less training parameters. This is one reason why the reviewed studies are mainly based on the CNN architecture.

#### Electronic health records

Electronic health records (EHRs) are systematic collections of longitudinal, patient-centered records. Patients’ EHRs consist of both structured and unstructured data: the structured data include information about a patient’s diagnosis, medications, and laboratory test results, and the unstructured data include information in clinical notes. Recently, DL models have been applied to analyze EHR data to study mental health disorders^48^.

The first and foremost issue for analyzing the structured EHR data is how to appropriately handle the longitudinal records. Traditional ML models address this by collapsing patients’ records within a certain time window into vectors, which comprised the summary of statistics of the features in different dimensions^49^. For instance, to estimate the probability of suicide deaths, Choi et al.^50^ leveraged a DFNN to model the baseline characteristics. One major limitation of these studies is the omittance of temporality among the clinical events within EHRs. To overcome this issue, RNNs are more commonly used for EHR data analysis as an RNN intuitively handles time-series data. DeepCare^51^, a long short-term memory network (LSTM)-based DL model, encodes patient’s long-term health state trajectories to predict the future outcomes of depressive episodes. As the LSTM architecture appropriately captures disease progression by modeling the illness history and the medical interventions, DeepCare achieved over 15% improvement in prediction, compared with the conventional ML methods. In addition, Lin et al.^52^ designed two DFNN models for the prediction of antidepressant treatment response and remission. The authors reported that the proposed DFNN can achieve an area under the receiver operating characteristic curve (AUC) of 0.823 in predicting antidepressant response.

Analyzing the unstructured clinical notes in EHRs refers to the long-standing topic of NLP. To extract meaningful knowledge from the text, conventional NLP approaches mostly define rules or regular expressions before the analysis. However, it is challenging to enumerate all possible rules or regular expressions. Due to the recent advance of DL in NLP tasks, DL models have been developed to mine clinical text data from EHRs to study mental health conditions. Geraci et al.^53^ utilized term frequency-inverse document frequency to represent the clinical documents by words and developed a DFNN model to identify individuals with depression. One major limitation of such an approach is that the semantics and syntax of sentences are lost. In this context, CNN^54^ and RNN^55^ have shown superiority in modeling syntax for text-based prediction. In particular, CNN has been used to mine the neuropsychiatric notes for predicting psychiatric symptom severity^56,57^. Tran and Kavuluru^58^ used an RNN to analyze the history of present illness in neuropsychiatric notes for predicting mental health conditions. The model engaged an attention mechanism^55^, which can specify the importance of the words in prediction, making the model more interpretable than their previous CNN model^56^.

##### Challenges and opportunities

Although DL has achieved promising results in EHR analysis, several challenges remain unsolved. On one hand, different from diagnosing physical health condition such as diabetes, the diagnosis of mental health conditions lacks direct quantitative tests, such as a blood chemistry test, a buccal swab, or urinalysis. Instead, the clinicians evaluate signs and symptoms through patient interviews and questionnaires during which they gather information based on patient’s self-report. Collection and deriving inferences from such data deeply relies on the experience and subjectivity of the clinician. This may account for signals buried in noise and affect the robustness of the DL model. To address this challenge, a potential way is to comprehensively integrate multimodal clinical information, including structured and unstructured EHR information, as well as neuroimaging and EEG data. Another way is to incorporate existing medical knowledge, which can guide model being trained in the right direction. For instance, the biomedical knowledge bases contain massive verified interactions between biomedical entities, e.g., diseases, genes, and drugs ^59^. Incorporating such information brings in meaningful medical constraints and may help to reduce the effects of noise on model training process. On the other hand, implementing a DL model trained from one EHR system into another system is challenging, because EHR data collection and representation is rarely standardized across hospitals and clinics. To address this issue, national/international collaborative efforts such as Observational Health Data Sciences and Informatics (https://ohdsi.org) have developed common data models, such as OMOP, to standardize EHR data representation for conducting observational data analysis^60^.

#### Genetic data

Multiple studies have found that mental disorders, e.g., depression, can be associated with genetic factors^61,62^. Conventional statistical studies in genetics and genomics, such as genome-wide association studies, have identified many common and rare genetic variants, such as single-nucleotide polymorphisms (SNPs), associated with mental health disorders^63,64^. Yet, the effect of the genetic factors is small and many more have not been discovered. With the recent developments in next-generation sequencing techniques, a massive volume of high-throughput genome or exome sequencing data are being generated, enabling researchers to study patients with mental health disorders by examining all types of genetic variations across an individual’s genome. In recent years, DL^65,66^ has been applied to identify genetic risk factors associated with mental illness, by borrowing the capacity of DL in identifying highly complex patterns in large datasets. Khan and Wang^67^ integrated genetic annotations, known brain expression quantitative trait locus, and enhancer/promoter peaks to generate feature vectors of variants, and developed a DFNN, named ncDeepBrain, to prioritized non-coding variants associated with mental disorders. To further prioritize susceptibility genes, they designed another deep model, iMEGES^68^, which integrates the ncDeepBrain score, general gene scores, and disease-specific scores for estimating gene risk. Wang et al.^69^ developed a novel deep architecture that combines deep Boltzmann machine architecture^70^ with conditional and lateral connections derived from the gene regulatory network. The model provided insights about intermediate phenotypes and their connections to high-level phenotypes (disease traits). Laksshman et al.^71^ used exome sequencing data to predict bipolar disorder outcomes of patients. They developed a CNN and used the convolution mechanism to capture correlations of the neighboring loci within the chromosome.

##### Challenges and opportunities

Although the use of genetic data in DL in studying mental health conditions shows promise, multiple challenges need to be addressed. For DL-based risk c/gene prioritization efforts, one major challenge is the limitation of labeled data. On one hand, the positive samples are limited, as known risk SNPs or genes associated with mental health conditions are limited. For example, there are about 108 risk loci that were genome-wide significant in ASD. On the other hand, the negative samples (i.e., SNPs, variants, or genes) may not be the “true” negative, as it is unclear whether they are associated with the mental illness yet. Moreover, it is also challenging to develop DL models for analyzing patient’s sequencing data for mental illness prediction, as the sequencing data are extremely high-dimensional (over five million SNPs in the human genome). More prior domain knowledge is needed to guide the DL model extracting patterns from the high-dimensional genomic space.

#### Vocal and visual expression data

The use of vocal (voice or speech) and visual (video or image of facial or body behaviors) expression data has gained the attention of many studies in mental health disorders. Modeling the evolution of people’s emotional states from these modalities has been used to identify mental health status. In essence, the voice data are continuous and dense signals, whereas the video data are sequences of frames, i.e., images. Conventional ML models for analyzing such types of data suffer from the sophisticated feature extraction process. Due to the recent success of applying DL in computer vision and sequence data modeling, such models have been introduced to analyze the vocal and/or visual expression data. In this work, most articles reviewed are to predict mental health disorders based on two public datasets: (i) the Chi-Mei corpus, collected by using six emotional videos to elicit facial expressions and speech responses of the subjects of bipolar disorder, unipolar depression, and healthy controls;^72^ and (ii) the International Audio/Visual Emotion Recognition Challenges (AVEC) depression dataset^73–75^, collected within human–computer interaction scenario. The proposed models include CNNs, RNNs, autoencoders, as well as hybrid models based on the above ones. In particular, CNNs were leveraged to encode the temporal and spectral features from the voice signals^76–80^ and static facial or physical expression features from the video frames^79,81–84^. Autoencoders were used to learn low-dimensional representations for people’s vocal^85,86^ and visual expression^87,88^, and RNNs were engaged to characterize the temporal evolution of emotion based on the CNN-learned features and/or other handcraft features^76,81,84–90^. Few studies focused on analyzing static images using a CNN architecture to predict mental health status. Prasetio et al.^91^ identified the stress types (e.g., neutral, low stress, and high stress) from facial frontal images. Their proposed CNN model outperforms the conventional ML models by 7% in terms of prediction accuracy. Jaiswal et al.^92^ investigated the relationship between facial expression/gestures and neurodevelopmental conditions. They reported accuracy over 0.93 in the diagnostic prediction of ADHD and ASD by using the CNN architecture. In addition, thermal images that track persons’ breathing patterns were also fed to a deep model to estimate psychological stress level (mental overload)^93^.

##### Challenges and opportunities

From the above summary, we can observe that analyzing vocal and visual expression data can capture the pattern of subjects’ emotion evolution to predict mental health conditions. Despite the promising initial results, there remain challenges for developing DL models in this field. One major challenge is to link vocal and visual expression data with the clinical data of patients, given the difficulties involved in collecting such expression data during clinical practice. Current studies analyzed vocal and visual expression over individual datasets. Without clinical guidance, the developed prediction models have limited clinical meanings. Linking patients’ expression information with clinical variables may help to improve both the interpretability and robustness of the model. For example, Gupta et al.^94^ designed a DFNN for affective prediction from audio and video modalities. The model incorporated depression severity as the parameter, linking the effects of depression on subjects’ affective expressions. Another challenge is the limitation of the samples. For example, the Chi-Mei dataset contains vocal–visual data from only 45 individuals (15 with bipolar disorder, 15 with unipolar disorder, and 15 healthy controls). Also, there is a lack of “emotion labels” for people’s vocal and visual expression. Apart from improving the datasets, an alternative way to solve this challenge is to use transfer learning, which transfers knowledge gained with one dataset (usually more general) to the target dataset. For example, some studies trained autoencoder in public emotion database such as eNTERFACE^95^ to generate emotion profiles (EPs). Other studies^83,84^ pre-trained CNN over general facial expression datasets^96,97^ for extracting face appearance features.

#### Social media data

With the widespread proliferation of social media platforms, such as Twitter and Reddit, individuals are increasingly and publicly sharing information about their mood, behavior, and any ailments one might be suffering. Such social media data have been used to identify users’ mental health state (e.g., psychological stress and suicidal ideation)^6^.

In this study, the articles that used DL to analyze social media data mainly focused on stress detection^98–101^, depression identification^102–106^, and estimation of suicide risk^103,105,107–109^. In general, the core concept across these work is to mine the textual, and where applicable graphical, content of users’ social media posts to discover cues for mental health disorders. In this context, the RNN and CNN were largely used by the researchers. Especially, RNN usually introduces an attention mechanism to specify the importance of the input elements in the classification process^55^. This provides some interpretability for the predictive results. For example, Ive et al.^103^ proposed a hierarchical RNN architecture with an attention mechanism to predict the classes of the posts (including depression, autism, suicidewatch, anxiety, etc.). The authors observed that, benefitting from the attention mechanism, the model can predict risk text efficiently and extract text elements crucial for making decisions. Coppersmith et al.^107^ used LSTM to discover quantifiable signals about suicide attempts based on social media posts. The proposed model can capture contextual information between words and obtain nuances of language related to suicide.

Apart from text, users also post images on social media. The properties of the images (e.g., color theme, saturation, and brightness) provide some cues reflecting users’ mental health status. In addition, millions of interactions and relationships among users can reflect the social environment of individuals that is also a kind of risk factors for mental illness. An increasing number of studies attempted to combine these two types of information with text content for predictive modeling. For example, Lin et al.^99^ leveraged the autoencoder to extract low-level and middle-level representations from texts, images, and comments based on psychological and art theories. They further extended their work with a hybrid model based on CNN by integrating post content and social interactions^101^. The results provided an implication that the social structure of the stressed users’ friends tended to be less connected than that of the users without stress.

##### Challenges and opportunities

The aforementioned studies have demonstrated that using social media data has the potential to detect users with mental health problems. However, there are multiple challenges towards the analysis of social media data. First, given that social media data are typically de-identified, there is no straightforward way to confirm the “true positives” and “true negatives” for a given mental health condition. Enabling the linkage of user’s social media data with their EHR data—with appropriate consent and privacy protection—is challenging to scale, but has been done in a few settings^110^. In addition, most of the previous studies mainly analyzed textual and image data from social media platforms, and did not consider analyzing the social network of users. In one study, Rosenquist et al.^111^ reported that the symptoms of depression are highly correlated inside the circle of friends, indicating that social network analysis is likely to be a potential way to study the prevalence of mental health problems. However, comprehensively modeling text information and network structure remains challenging. In this context, graph convolutional networks^112^ have been developed to address networked data mining. Moreover, although it is possible to discover online users with mental illness by social media analysis, translation of this innovation into practical applications and offer aid to users, such as providing real-time interventions, are largely needed^113^.

## Discussion: findings, open issues, and future directions

### Principle findings

The purpose of this study is to investigate the current state of applications of DL techniques in studying mental health outcomes. Out of 2261 articles identified based on our search terms, 57 studies met our inclusion criteria and were reviewed. Some studies that involved DL models but did not highlight the DL algorithms’ features on analysis were excluded. From the above results, we observed that there are a growing number of studies using DL models for studying mental health outcomes. Particularly, multiple studies have developed disease risk prediction models using both clinical and non-clinical data, and have achieved promising initial results.

### Data bias

DL models “think to learn” like a human brain relying on their multiple layers of interconnected computing neurons. Therefore, to train a deep neural network, there are multiple parameters (i.e., weights associated links between neurons within the network) being required to learn. This is one reason why DL has achieved great success in the fields where a massive volume of data can be easily collected, such as computer vision and text mining. Yet, in the health domain, the availability of large-scale data is very limited. For most selected studies in this review, the sample sizes are under a scale of 10^4^. Data availability is even more scarce in the fields of neuroimaging, EEG, and gene expression data, as such data reside in a very high-dimensional space. This then leads to the problem of “curse of dimensionality”^114^, which challenges the optimization of the model parameters.

One potential way to address this challenge is to reduce the dimensionality of the data by feature engineering before feeding information to the DL models. On one hand, feature extraction approaches can be used to obtain different types of features from the raw data. For example, several studies reported in this review have attempted to use preprocessing tools to extract features from neuroimaging data. On the other hand, feature selection that is commonly used in conventional ML models is also an option to reduce data dimensionality. However, the feature selection approaches are not often used in the DL application scenario, as one of the intuitive attributes of DL is the capacity to learn meaningful features from “all” available data. The alternative way to address the issue of data bias is to use transfer learning where the objective is to improve learning a new task through the transfer of knowledge from a related task that has already been learned^115^. The basic idea is that data representations learned in the earlier layers are more general, whereas those learned in the latter layers are more specific to the prediction task^116^. In particular, one can first pre-train a deep neural network in a large-scale “source” dataset, then stack fully connected layers on the top of the network and fine-tune it in the small “target” dataset in a standard backpropagation manner. Usually, samples in the “source” dataset are more general (e.g., general image data), whereas those in the “target” dataset are specific to the task (e.g., medical image data). A popular example of the success of transfer learning in the health domain is the dermatologist-level classification of skin cancer^117^. The authors introduced Google’s Inception v3 CNN architecture pre-trained over 1.28 million general images and fine-tuned in the clinical image dataset. The model achieved very high-performance results of skin cancer classification in epidermal (AUC = 0.96), melanocytic (AUC = 0.96), and melanocytic–dermoscopic images (AUC = 0.94). In facial expression-based depression prediction, Zhu et al.^83^ pre-trained CNN on the public face recognition dataset to model the static facial appearance, which overcomes the issue that there is no facial expression label information. Chao et al.^84^ also pre-trained CNN to encode facial expression information. The transfer scheme of both of the two studies has been demonstrated to be able to improve the prediction performance.

### Diagnosis and prediction issues

Unlike the diagnosis of physical conditions that can be based on lab tests, diagnoses of the mental illness typically rely on mental health professionals’ judgment and patient self-report data. As a result, such a diagnostic system may not accurately capture the psychological deficits and symptom progression to provide appropriate therapeutic interventions^118,119^. This issue accordingly accounts for the limitation of the prediction models to assist clinicians to make decisions. Except for several studies using the unsupervised autoencoder for learning low-dimensional representations, most studies reviewed in this study reported using supervised DL models, which need the training set containing “true” (i.e., expert provided) labels to optimize the model parameters before the model being used to predict labels of new subjects. Inevitably, the quality of the expert-provided diagnostic labels used for training sets the upper-bound for the prediction performance of the model.

One intuitive route to address this issue is to use an unsupervised learning scheme that, instead of learning to predict clinical outcomes, aims at learning compacted yet informative representations of the raw data. A typical example is the autoencoder (as shown in Fig. 1d), which encodes the raw data into a low-dimensional space, from which the raw data can be reconstructed. Some studies reviewed have proposed to leverage autoencoder to improve our understanding of mental health outcomes. A constraint of the autoencoder is that the input data should be preprocessed to vectors, which may lead to information loss for image and sequence data. To address this, recently convolutional-autoencoder^120^ and LSTM-autoencoder^121^ have been developed, which integrate the convolution layers and recurrent layers with the autoencoder architecture and enable us to learn informative low-dimensional representations from the raw image data and sequence data, respectively. For instance, Baytas et al.^122^ developed a variation of LSTM-autoencoder on patient EHRs and grouped Parkinson’s disease patients into meaningful subtypes. Another potential way is to predict other clinical outcomes instead of the diagnostic labels. For example, several selected studies proposed to predict symptom severity scores^56,57,77,82,84,87,89^. In addition, Du et al.^108^ attempted to identify suicide-related psychiatric stressors from users’ posts on Twitter, which plays an important role in the early prevention of suicidal behaviors. Furthermore, training model to predict future outcomes such as treatment response, emotion assessments, and relapse time is also a promising future direction.

### Multimodal modeling

**Fig. 4:**
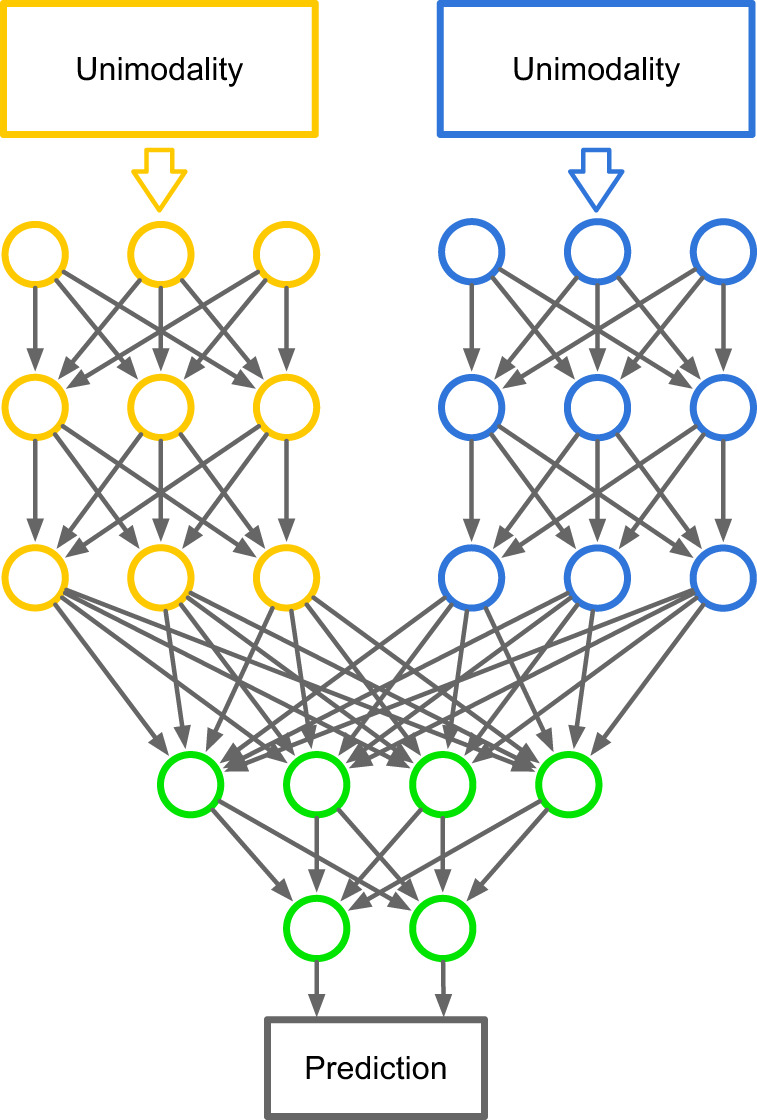
An illustration of the multimodal deep neural network. One can model each modality with a specific network and combine them using the final fully-connected layers. In this way, parameters of the entire neural network can be jointly learned in a typical backpropagation manner.

The field of mental health is heterogeneous. On one hand, mental illness refers to a variety of disorders that affect people’s emotions and behaviors. On the other hand, though the exact causes of most mental illnesses are unknown to date, it is becoming increasingly clear that the risk factors for these diseases are multifactorial as multiple genetic, environmental, and social factors interact to influence an individual’s mental health^123,124^. As a result of domain heterogeneity, researchers have the chance to study the mental health problems from different perspectives, from molecular, genomic, clinical, medical imaging, physiological signal to facial, and body expressive and online behavioral. Integrative modeling of such multimodal data means comprehensively considering different aspects of the disease, thus likely obtaining deep insight into mental health. In this context, DL models have been developed for multimodal modeling. As shown in Fig. 4, the hierarchical structure of DL makes it easily compatible with multimodal integration. In particular, one can model each modality with a specific network and combine them by the final fully connected layers, such that parameters can be jointly learned by a typical backpropagation manner. In this review, we found an increasing number of studies have attempted to use multimodal modeling. For example, Zou et al.^28^ developed a multimodal model composed of two CNNs for modeling fMRI and sMRI modalities, respectively. The model achieved 69.15% accuracy in predicting ADHD, which outperformed the unimodal models (66.04% for fMRI modal-based and 65.86% for sMRI modal-based). Yang et al.^79^ proposed a multimodal model to combine vocal and visual expression for depression cognition. The model results in 39% lower prediction error than the unimodal models.

### Model interpretability

Due to the end-to-end design, the DL models usually appear to be “black boxes”: they take raw data (e.g., MRI images, free-text of clinical notes, and EEG signals) as input, and yield output to reach a conclusion (e.g., the risk of a mental health disorder) without clear explanations of their inner working. Although this might not be an issue in other application domains such as identifying animals from images, in health not only the model’s prediction performance but also the clues for making the decision are important. For example in the neuroimage-based depression identification, despite estimation of the probability that a patient suffers from mental health deficits, the clinicians would focus more on recognizing abnormal regions or patterns of the brain associated with the disease. This is really important for convincing the clinical experts about the actions recommended from the predictive model, as well as for guiding appropriate interventions. In addition, as discussed above, the introduction of multimodal modeling leads to an increased challenge in making the models more interpretable. Attempts have been made to open the “black box” of DL^59,125–127^. Currently, there are two general directions for interpretable modeling: one is to involve the systematic modification of the input and the measure of any resulting changes in the output, as well as in the activation of the artificial neurons in the hidden layers. Such a strategy is usually used in CNN in identifying specific regions of an image being captured by a convolutional layer^128^. Another way is to derive tools to determine the contribution of one or more features of the input data to the output. In this case, the widely used tools include Shapley Additive Explanation^129^, LIME^127^, DeepLIFT^130^, etc., which are able to assign each feature an importance score for the specific prediction task.

### Connection to therapeutic interventions

According to the studies reviewed, it is now possible to detect patients with mental illness based on different types of data. Compared with the traditional ML techniques, most of the reviewed DL models reported higher prediction accuracy. The findings suggested that the DL models are likely to assist clinicians in improved diagnosis of mental health conditions. However, to associate diagnosis of a condition with evidence-based interventions and treatment, including identification of appropriate medication^131^, prediction of treatment response^52^, and estimation of relapse risk^132^ still remains a challenge. Among the reviewed studies, only one^52^ proposed to target at addressing these issues. Thus, further efforts are needed to link the DL techniques with the therapeutic intervention of mental illness.

### Domain knowledge

Another important direction is to incorporate domain knowledge. The existing biomedical knowledge bases are invaluable sources for solving healthcare problems^133,134^. Incorporating domain knowledge could address the limitation of data volume, problems of data quality, as well as model generalizability. For example, the unified medical language system^135^ can help to identify medical entities from the text and gene–gene interaction databases^136^ could help to identify meaningful patterns from genomic profiles.

## Conclusion

Recent years have witnessed the increasing use of DL algorithms in healthcare and medicine. In this study, we reviewed existing studies on DL applications to study mental health outcomes. All the results available in the literature reviewed in this work illustrate the applicability and promise of DL in improving the diagnosis and treatment of patients with mental health conditions. Also, this review highlights multiple existing challenges in making DL algorithms clinically actionable for routine care, as well as promising future directions in this field.

## Supplementary information

Supplemental Material
