# Supplementary material for: Deep learning in mental health outcome research: a scoping review
Source: Transl Psychiatry. 2020 Apr 22;10:116. doi: 10.1038/s41398-020-0780-3 (PMC7293215; doi:10.1038/s41398-020-0780-3)
Supplement: Supplementary file 1 — Supplemental Material [file 41398_2020_780_MOESM1_ESM.docx]

**Supplementary of Deep learning in mental health: A scoping review**

Appendix 1: Queries

**Pubmed:**

(mental health[Title/Abstract]) OR (mental health[Text Word]) OR (mental disorder*[Text Word]) OR (mental illness[Text Word]) OR (mental condition*[Text Word]) OR (psychologic*[Text Word]) OR (psychology[Text Word]) OR (psychiatry*[Text Word]) OR (psychiatric*[Text Word]) OR (psychotic disorder*[Text Word]) OR (depressive disorder*[Text Word]) OR (depression[Text Word]) OR (suicide*[Text Word]) OR (suicidal[Text Word]) OR (anxiety[Text Word]) OR (mood disorder*[Text Word]) OR (personality[Text Word]) OR (ADHD[Text Word]) OR (developmental disorder*[Text Word])) AND ((deep learning[Title/Abstract]) OR (deep learning[Text Word]) OR (deep neural network*[Title/Abstract]) OR (deep neural network*[Text Word]) OR (recurrent neural network*[Text Word]) OR (convolutional neural network*[Text Word]) OR (autoencoder[Text Word]) OR (artificial neural network*[Text Word]) OR (artificial intelligence[Text Word]) OR (deep machine learning[Text Word]))

**Web of science:**

#1: TS="mental health" OR TS="mental disorder*" OR TS="mental illness" OR TS="mental condition*"

#2: TS="psychologic*" OR TS="psychology" OR TS="psychiatry*" OR TS="psychiatric*" OR TS="psychotic disorder*" OR TS="depressive disorder*" OR TS="depression" OR TS="suicide*" OR TS="suicidal" OR TS="anxiety" OR TS="mood disorder*" OR TS="personality" OR TS="ADHD" OR TS="developmental disorder*"

#3: TS="deep learning" OR TS="deep neural network*" OR TS="recurrent neural network*" OR TS="RNN" OR TS="convolutional neural network*" OR TS= "CNN" OR TS="autoencoder" OR TS="artificial neural network*" OR TS="artificial intelligence" OR TS="deep machine learning"

#4: (#1 OR #2) AND #3

Indexes=SCI-EXPANDED, SSCI, A&HCI, CPCI-S, CPCI-SSH, BKCI-S, BKCI-SSH, ESCI Timespan=All years
